# Perinatal exposure to tributyltin affects feeding behavior and expression of hypothalamic neuropeptide Y in the paraventricular nucleus of adult mice

**DOI:** 10.1111/joa.13766

**Published:** 2022-09-08

**Authors:** Giovanna Ponti, Elisabetta Bo, Brigitta Bonaldo, Alice Farinetti, Marilena Marraudino, Giancarlo Panzica, Stefano Gotti

**Affiliations:** ^1^ Neuroscience Institute Cavalieri Ottolenghi (NICO) Orbassano Italy; ^2^ Department of Neuroscience “Rita Levi‐Montalcini” University of Torino Torino Italy

**Keywords:** development, endocrine disrupting chemicals, feeding behavior, hypothalamic circuits, NPY, paraventricular hypothalamic nucleus, sexual dimorphism

## Abstract

Organotins such as tributyltin chloride (TBT), are highly diffused environmental pollutants, which act as metabolism disrupting chemicals, i.e. may interfere with fat tissue differentiation, as well as with neuroendocrine circuits, thus impairing the control of energetic balance. We have previously demonstrated that adult exposure to TBT altered the expression of neuropeptides in the hypothalamus. In this study, we orally administered daily a solution containing oil, or TBT (0.25, 2.5, or 25 μg/kg body weight/day) to pregnant females from gestational day 8 until birth, and to their pups from day 0 until post‐natal day 21. Our results showed that TBT exposure of female mice during gestation and of pups during lactation permanently altered the feeding efficiency of pups of both sexes and subcutaneous fat distribution in adult males. In addition, the neuropeptide Y system was affected at the level of the paraventricular nucleus, with a decrease in immunoreactivity in both sexes (significant in females for all TBT doses and in males only for intermediate TBT doses), while no effect was observed in other hypothalamic areas (arcuate, ventromedial and dorsomedial nuclei). Metabolic syndrome, as well as obesity and diabetes, which are significant health issues, are considered multifactorial diseases and may be caused by exposure to metabolic disruptors, both in adults and during perinatal life. In addition, our work indicates that TBT doses defined as the tolerably daily intake had a profound and sex‐specific long‐term effect.

## Graphical abstract

We examined the effect of oral administration of tributyltin (TBT) to dams and pups. Our results show that TBT exposure alters feeding efficiency and subcutaneous fat distribution. In addition, we found an alteration of the neuropeptide Y system at the level of the paraventricular nucleus.

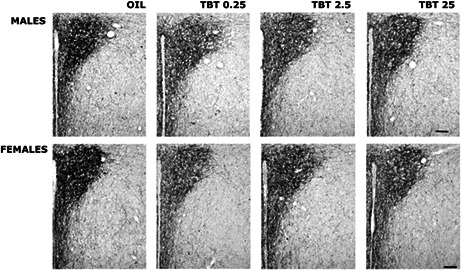

## 1 INTRODUCTION

Over the last two decades, the incidence of obesity and associated metabolic syndromes has dramatically risen, becoming a global health crisis both for humans and for many other species (Klimentidis et al., 2011). While, increased caloric intake and decreased physical activity are the leading causes of metabolic disorders, recent findings highlighted the possible involvement of environmental metabolism disrupting chemicals (MDCs; Heindel et al., 2015, 2017). MDCs are xenobiotic chemicals that disrupt normal developmental and homeostatic controls of adipogenesis and energy balance (Heindel & Blumberg, 2019). Among them, organotins are a class of widespread persistent pollutants with high endocrine‐disrupting activity, both in vertebrates and invertebrates (for detailed reviews see Ferraz da Silva et al., 2018, Grun, 2014). Organotins, such as tributyltin chloride (TBT), are widely used as biocides in ships and fishing nets, agricultural fungicides, and rodent repellents. They are common contaminants in marine and freshwater ecosystems exceeding acute and chronic toxicity levels (Nakanishi, 2008). Despite a ban on many TBT‐based products, which led to a decrease in TBT levels in the water, these are still high in sediments (Abraham et al., 2017; Egardt et al., 2017). Significant TBT levels were also observed in humans (Antizar‐Ladislao, 2008). TBT is a potent inducer of adipogenesis, both in vitro and in vivo, as shown in newborn mice after intrauterine TBT exposure (Grun et al., 2006). Moreover, peripubertal chronic exposure to TBT (starting from 5 μg/kg body weight) induced a dose‐dependent increase in body weight of young male mice. At this dose, mice displayed increased circulating levels of insulin, leptin and hepatic resistin compared to the control group (Zuo et al., 2009). Furthermore, TBT may have an obesogenic effect mediated through the microbiota (Guo et al., 2018). Thus, TBT is an MDC that acts peripherally on adipogenesis, but metabolic dysregulation may also depend on a malfunction of neuroendocrine brain circuits involved in the control of energetic metabolism. In fact, feeding behaviour is regulated by the peripheral signals regarding nutritional status directed to the central nervous system (Gao & Horvath, 2007; Morton et al., 2006). Indeed, plasma levels of leptin are elevated in individuals with obesity, although development of obesity is probably due to defective leptin transport to the central nervous system or due to leptin resistance (Myers et al., 2008). For example, after lesions in ventromedial hypothalamic nucleus (VMH), leptin production is increased concomitant with reduced availability of neuropeptide Y (NPY) in the paraventricular hypothalamic nucleus (PVN) and loss of the regulated daily pattern of feeding. The resultant hyperphagia may be a consequence of rapid development of NPY receptor super‐sensitivity (Kalra et al., 1998). NPY is one of the most potent orexigenic peptides: chronic administration of NPY in the brain promotes hyperphagia and body weight gain in rodents. Therefore, activation of the NPY‐NPY receptors pathway is critical for obesity development (Nguyen et al., 2011; Sato et al., 2009).

We have previously demonstrated that acute TBT exposure stimulated the arcuate nucleus (ARC) (Bo et al., 2011) and that chronic TBT treatment in adults (4 weeks, or 16 weeks) affected the leptin‐NPY‐Y1 receptor axis and Pro‐Opio‐Melanocortin (POMC) expression (Bo et al., 2016; Farinetti et al., 2018; Marraudino et al., 2021). Similar studies demonstrated the involvement of the hypothalamus‐hypophyseal‐thyroid axis (Andrade et al., 2018; Santos‐Silva et al., 2018). Late gestation and early postnatal life are particularly sensitive periods for the development of many neuroendocrine circuits and perturbations of the hormonal environment during these periods may lead to permanent alterations of neural circuits and behaviours (Panzica & Melcangi, 2016). However, to our knowledge, only a few studies have examined the hypothalamic effects of perinatal exposure to TBT (i.e. on the hypothalamus‐hypophysis‐thyroid axis, Decherf et al., 2010), and none has investigated whether precocious exposure to TBT may induce permanent alterations in hypothalamic circuits directly related to feeding behaviour and the control of energy metabolism. Thus, the aim of this study was to understand whether perinatal TBT exposure permanently alters hypothalamic NPY expression and the regulation of feeding behaviour.

## 2 MATERIAL AND METHODS

### 2.1 Animals and treatment

Animal care and handling during experimental procedures were in accordance with the European Union Council Directive of 22nd September 2010 (2010/63/UE); the Italian Ministry of Health and the Ethical Committee of the University of Torino approved the procedures reported in the present study. Twenty 6‐week‐old virgin C57/BL6 female mice and ten 6‐week‐old virgin C57/BL6 male mice were purchased from HARLAN Italy, housed in monosexual groups of 5 animals in 45 × 25 × 15 cm polypropylene mouse cages at 22 ± 1°C under a photoperiod of 12 h dark/light, with water and food ad libitum. After a 2‐weeks adaptation period in the animal house, two females were housed with one male per cage, until a vaginal plug was detected (defined as day 0 of gestation). After mating, pregnant females were housed individually and were randomly allocated to four groups of treatments: OIL, (mice receiving only olive oil as vehicle, purchased from Sigma‐Aldrich, Europ, 0514), TBT 0.25, TBT 2.5 and TBT 25 (mice receiving 0.25, 2.5, 25 μg/kg of body weight/day TBT, respectively; stock solution 96% from Sigma‐Aldrich, Europe, T50202). A total of 30 μl TBT diluted in oil in the indicated concentrations was administered orally in each animal. TBT solutions were prepared weekly.

Given the high toxicity of TBT in adult mice reported in our previous study (Bo et al., 2016), the higher dose used here corresponded to the lower dose used for the treatment of adult mice in our previous experiments. In particular, according to the literature, the higher dose is a ‘no observable adverse effect dose’ (NOAEL) and the lower dose corresponded to the ‘tolerable daily intake’ (TDI) dose (Penninks, 1993).

**FIGURE 1:**
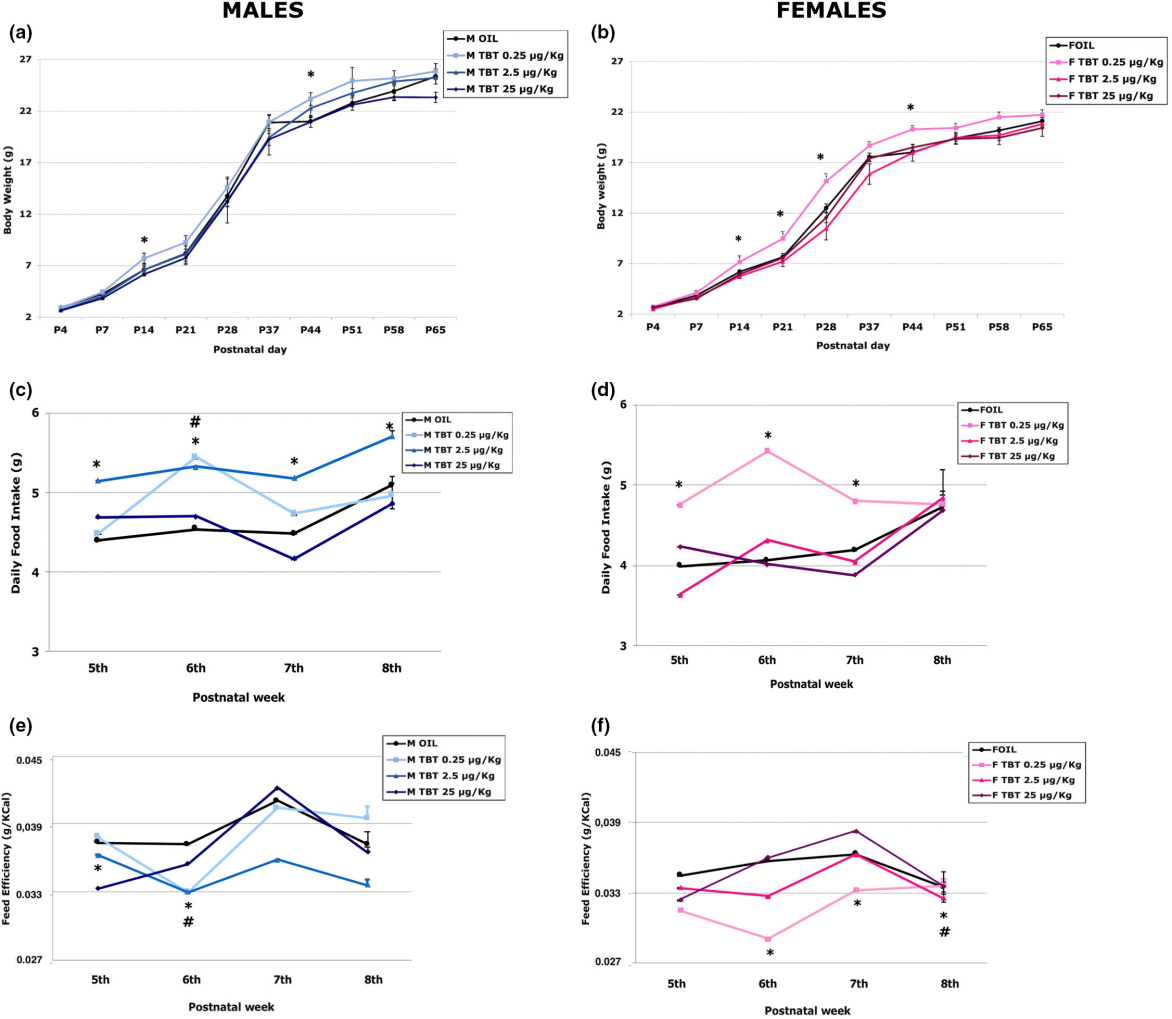
Effect of TBT exposure on energetic metabolism. (A) The lines represent variations of body weight (in grams) in male mice during the experiment from P0 to P65. With the dose of 0.25 there is a significant increase in comparison to controls (OIL) at P14 and during the week 6–7. (B) The lines represent variations of body weight (in grams) in female mice during the experiment from P0 to P65. The lower dose of TBT (0.25) induced the increase of body weight during the post‐natal life, at P14, P21, P28 and during the week 6. (C) The lines represent variations of the daily food intake from the 5th to the 8th week in male mice. There is a significant increase of food consumption in males treated with TBT 2.5 during the 4 weeks period considered. (D) The lines represent variations of the daily food intake from the 5th to the 8th week in female mice. There is a significant increase of food consumption in females treated with TBT 0.25 during the first 3 weeks considered. (E) The lines represent the variations of daily feeding efficiency (expressed as body weight/kcal assumed) in male mice. During all the considered period male mice showed a reduction of feeding efficiency; in particular, after treatment with TBT 2.5. (F) The lines represent the variations of daily feeding efficiency (expressed as body weight/kcal assumed) in female mice. The lower dose of TBT (0.25) induces the reduction of feeding efficiency; in particular, in week 6 and 7. While the intermediate dose (2.5 μg) induces reduction of feeding efficiency during the first 2 weeks of the considered period.

Solutions were orally administered daily to pregnant females from gestational day 8 (G8) until birth and to their pups from day 0 until post‐natal day 21 (P21; Figure 1a). Oral doses were administered with a micropipette as described previously (Bo et al., 2016; Palanza et al., 2001). Briefly, mice were picked up by the skin between the shoulders and held upright. The tip of a micropipette was placed into the mouth of mice and the solution was released from the pipette. Mice received approximately 30 μl of solution, adjusted according to body weight.

Pups were identified at birth by toe clipping. Pups were housed with their mother until weaning, and then housed in treatment‐differentiated monosexual groups of 3–5 animals, as described above.

In the present study we used totally 104 mice: 20 females and 10 males for the mating and 74 mice for analysis:

29 OIL, (13 males;16 females)

19 TBT 0.25, (10 males;9 females)

17 TBT 2.5 (5 males;12 females)

9 TBT 25, (5 males;4 females)

### 2.2 Body weight and food consumption

Offspring's body weight was measured with an electronic precision balance (Mod. Kern‐440, capacity 500 g and accuracy 1 mg) at P4, P7, P14, P21, P28 and from P29 until P60 twice a week (Figure 1a,b). Animals were fed with ad libitum access to water and to a standard diet 4RF21, GLP certificate (Mucedola, Italy) containing 2668 Kcal/g of metabolisable energy with 21.7% protein, 0.4% fat and 66.5% carbohydrates with a certificated estrogenic activity lower than 20 μg/kg of DES equivalent (Mucedola srl, Settimo Milanese, Italy).

Starting from 5 weeks of age until euthanasia (8 weeks), food consumption was measured once weekly. Food consumption per cage per week was calculated as the difference between the weight of food supplied and the weight of the residual food found in each cage. Food consumption for each mouse (mean grams per mouse per day) was calculated as the ratio of total food consumption in the cage divided by the number of animals. After measurement, mice were provided fresh quantity of food.

Daily energy intake was calculated by multiplying daily food intake by the caloric value of the chow (2668 Kcal/g). Daily feeding efficiency was expressed as body weight (g)/Kcal consumed (Michel et al., 2003).

### 2.3 Tissue sampling

All mice were killed at the age of 8 weeks. Females were inspected by daily examination of vaginal cytology smears (for details see McLean et al., 2012). Females in diestrus were used for immunohistochemical processing after exhibiting 2 or more consecutive 4‐days estrous cycles.

Male and female mice were deeply anesthetised with a mixture of ketamine‐xylazine (100 mg/ml and 20 mg/ml, respectively) and perfused through the heart with saline solution (0.9%) until vessels were completely blood‐free, followed by perfusion with 4% paraformaldehyde in 0.1 M phosphate buffer (pH 7.3), as fixative. After perfusion, livers and white fat pads were dissected (according to Cinti, 2001) and weighted, while brains were dissected and post‐fixed for 24 h in the same fixative at 4°C, rinsed several times with PBS, placed overnight at 4°C in PBS containing 30% sucrose solution until they sank. The next day, brains were frozen in isopentane pre‐cooled in dry ice at −30°C/−40°C, and finally stored at −80°C.

### 2.4 NPY immunohistochemistry

Brains were serially cut in the coronal plane with a cryostat (Leica CM 1900) at 25 μm of thickness, in four series. The plane of sectioning was oriented to match the drawings corresponding to the coronal sections of the mouse brain atlas (Paxinos & Franklin, 2001). Sections were collected in a cryoprotectant solution (Watson et al., 1986) and stored at −20°C.

One series of sections was Nissl‐stained with toluidine blue for anatomical reference. All structures were identified using the stereotaxic atlas of the mouse brain (Paxinos & Franklin, 2001).

Another series of sections was processed for NPY immunohistochemistry using the free‐floating technique. Brain sections were always stained in groups containing sections from male and female mice from all treatments, to minimise inter‐assay variance. Briefly, after overnight washing with PBS, sections were treated with Triton X‐100 (0.2% in PBS) for 30 min followed by methanol/hydrogen peroxide for 20 min at room temperature, to block endogenous peroxidase activity. Then, sections were incubated with normal goat serum (Vector Laboratories) for 30 min and with a rabbit polyclonal antibody against synthetic porcine NPY (a generous gift of H. Vaudry) diluted 1:6000 in PBS, pH 7.3–7.4, containing 0.2% Triton X‐100, overnight at room temperature. The following day, sections were incubated for 60 min with biotinylated goat anti‐rabbit IgG (Vector, Laboratories) at a dilution of 1:200 at room temperature. The antigen–antibody reaction was revealed by 60 min incubation with the biotin‐avidin system (BAS, Vectastain Elite kit, Vector Laboratories). Peroxidase activity was visualised with a solution containing 0.400 mg/ml 3,3′‐diamino‐benzidine (DAB, Sigma‐Aldrich, Europe) and 0.004% hydrogen peroxide in 0.05 M Tris–HCl buffer pH 7.6. Sections were mounted on chromalum‐coated slides, air‐dried, washed with xylene, and cover‐slipped with Entellan (Merck).

Specificity and cross‐reactivity of anti‐NPY antibody with other neuropeptides have been previously reported (Allen et al., 1983; Pelletier, Desy, et al., 1984a; Pelletier, Guy, et al., 1984b). This antibody was largely used for immunohistochemical detection of NPY in a wide range of vertebrate species, including rodents and humans (Danger et al., 1990). In addition, we performed the following negative controls to ensure antibody specificity: (a) we used an equivalent concentration of normal serum, instead of anti‐NPY; (b) the secondary antibody was omitted. In these conditions, cells and fibres were completely unstained.

### 2.5 Quantitative analysis

#### 2.5.1 Quantification of immunoreactivity

We examined the following hypothalamic nuclei involved in controlling feeding behaviour: arcuate nucleus (ARC), ventromedial hypothalamic nucleus (VMH), dorsomedial hypothalamic nucleus (DMH), and paraventricular hypothalamic nucleus (PVN). For each nucleus, we measured the density of NPY‐immunoreactive structures on three consecutive sections identified according to the Mouse Brain Atlas (Paxinos & Franklin, 2001; Arc, VMH, DMH: bregma −1.46 mm, −1.58 mm, −1.70 mm; PVN: bregma −0.70 mm, −0.82 mm, −0.94 mm). Sections were observed with a microscope Nikon eclipse 80i equipped with a Nikon digital sight camera DS‐Fi1; images for selected fields were then directly acquired. Staining density of NPY‐immunoreactive (ir) structures was measured in the selected nuclei with the Image J freeware software (Wayne Rasband, NIH, Bethesda, Maryland, USA) by calculating, in binary transformations of the images (threshold function), the fractional area (percentage of pixels) covered by immunoreactive structures in predetermined fields (area of interest, AOI) as described in previous studies (Bo et al., 2016; Marraudino et al., 2021). The AOI selected for each nucleus was a box of fixed size and shape, selected to cover immunoreactive material only within the boundaries of each nucleus (140,000 μm^2^ for VMH and DMH; 110,000 μm^2^ for ARC; and 200,000 μm^2^ for PVN). All the quantifications were performed by one individual (B.B.) blinded to treatment groups.

### 2.6 Statistical analysis

Quantitative data were examined with SPSS 24.0 statistic software (SPSS Inc.) by analysis of variance (two‐way ANOVA), where sex and treatment were considered independent variables. When appropriate, we performed a multivariate test (Tukey post‐hoc test) to compare between groups; differences between groups were considered significant for *p* ≤ 0.05.

## 3 RESULTS

### 3.1 Body weight, food intake, and feeding efficiency

Adult (Figure 1) TBT‐treated mice showed an increase in food consumption (Figure 1c,d). Two‐way ANOVA analysis (sex and treatments as independent variables) showed significant effects for both sex (*F*_[1]_ = 18.262 and *p* ≤ 0.001) and treatment (*F*_[3]_ = 7.405 and *p* ≤ 0.001), but no effects were observed for the interaction between the two variables (*F*_[1,3]_ = 2.089). Control males displayed higher food intake than control females in all weeks examined. TBT treatment increased food intake in both sexes, however with important differences. In males, the intermediate TBT dose (TBT 2.5 μg/kg of body weight/ day) permanently increased food consumption compared to control mice (globally *p* ≤ 0.05), while the effect of the lower TBT dose (0.25 μg/kg of body weight/day) was limited at the 6th week. In females, we observed a transient effect limited to the lower dose of TBT (globally *p* ≤ 0.05) in the first 3 weeks (Figure 1b).

In addition, the body weight was affected by TBT treatment (Figure 1a,b). In fact, two‐way ANOVA analysis showed significant differences for sex (*F*_[1]_ = 49.894 and *p* ≤ 0.001) and treatment (*F*_[3]_ = 3.087 and *p* ≤ 0.05), but no significant effect was detected for the interaction between sex and treatment (*F*_[1,3]_ = 0.303). However, only a minor effect was observed in adult animals with the lower dose of TBT: male animals showed an increase in body weight at P14 and during weeks 6–7; female animals showed an increase in body weight at P14, P21, P28 and during week 6 (males *p* ≤ 0.05; females *p* ≤ 0.010).

As a consequence of the alterations of these two parameters, feeding efficiency (the ratio between body weight gain and Kcal introduced by food, Michel et al., 2003), was also affected by TBT treatment (Figure 1e,f). Two‐way ANOVA analysis showed a statistically significant effect only for sex (*F*_[1]_ = 13.857 and *p* ≤ 0.001), while TBT treatment and the interaction between the two variables were not significant (*F*_(3)_ = 2.074 and *F*_(1,3)_ = 0.269). As expected (Bo et al., 2016), control males had a higher feeding efficiency than females.

### 3.2 White fat deposition and liver

**FIGURE 2:**
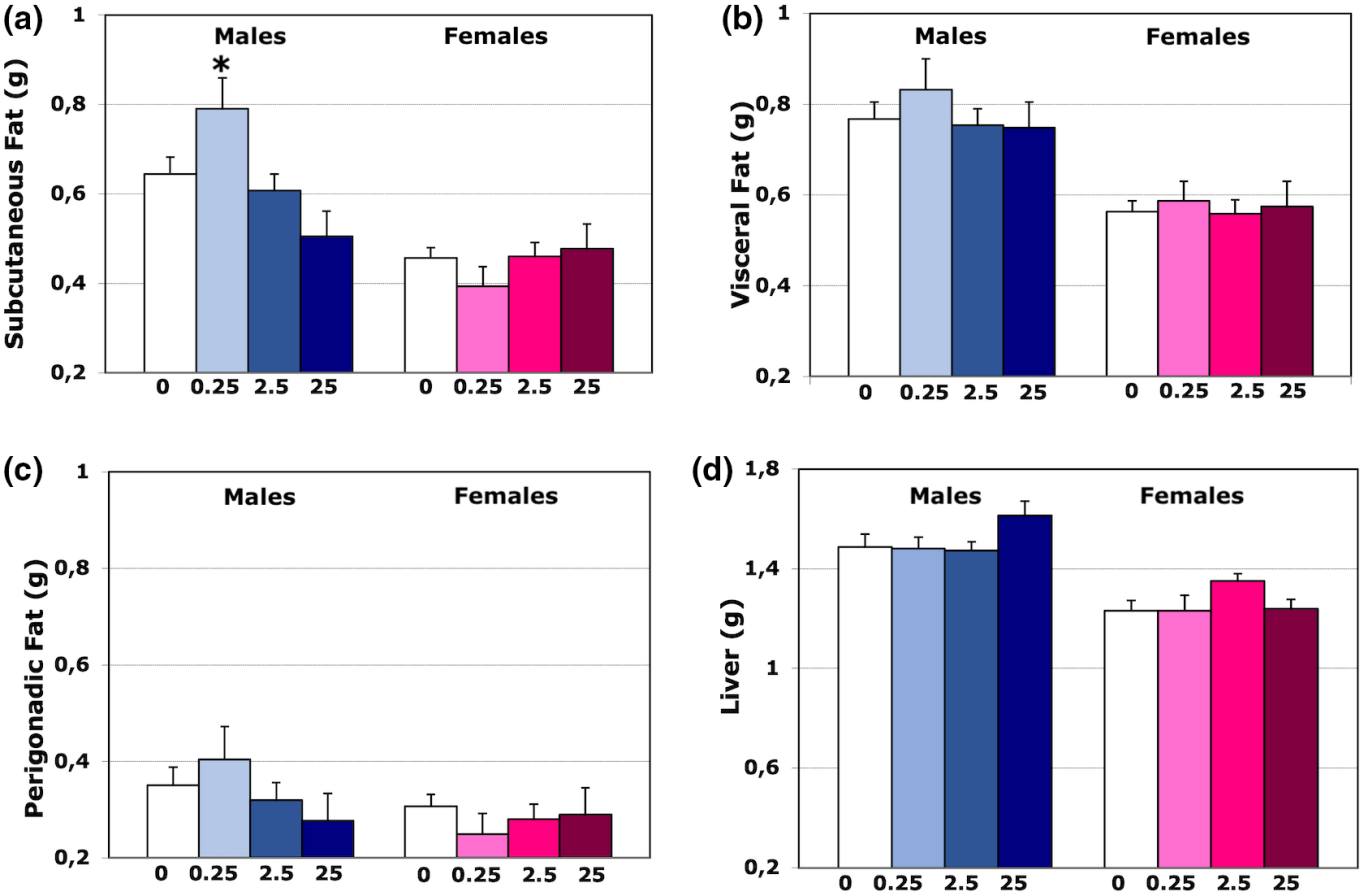
Effect of TBT exposure on fat and on liver weight. (A–C) Histograms represent subcutaneous (A), visceral (B), and perigonadal (C) fat deposition (expressed in grams). The only significant variation is in the subcutaneous fat of male mice treated with the lower dose of TBT (0.25) (A). (D) Histograms representing variations of the liver weight (in grams). There is a sexual difference in liver weight, with female livers smaller than male ones. No significant effect is observed in TBT treated animals.

Manually (Figure 2) dissected fat pads were separated (according to Cinti, 2001) in subcutaneous, perigonadic, and visceral fat and weighted. In controls mice, a sex difference was observed for subcutaneous (Figure 2a) and visceral fat (Figure 2b), whereas no sex difference was detected for perigonadic fat (Figure 2c). Sex differences were not altered by exposure to different TBT doses, as confirmed by two‐way ANOVA analysis showing significant effects of sex for subcutaneous and visceral fat (*F*_(1,66)_ = 19.905 and *p* ≤ 0.001, and *F*_(1,66)_ = 30.255 and *p* ≤ 0.001, respectively).

Liver weight and size were not affected by TBT treatments. Two‐way ANOVA analysis confirmed that the only effect on liver weight depended on sex (*F*_(1,66)_ = 39.199 and *p* ≤ 0.001), while no effects were found for treatment (*F*_[3,66]_ = 0.775) or for the interaction between sex and treatment (*F*_[3,66]_ = 1.241). In particular, in control mice, multiple comparison analysis showed that males had heavier livers than females (*p* ≤ 0.001; Figure 2d).

### 3.3 NPY immunoreactivity

**FIGURE 3:**
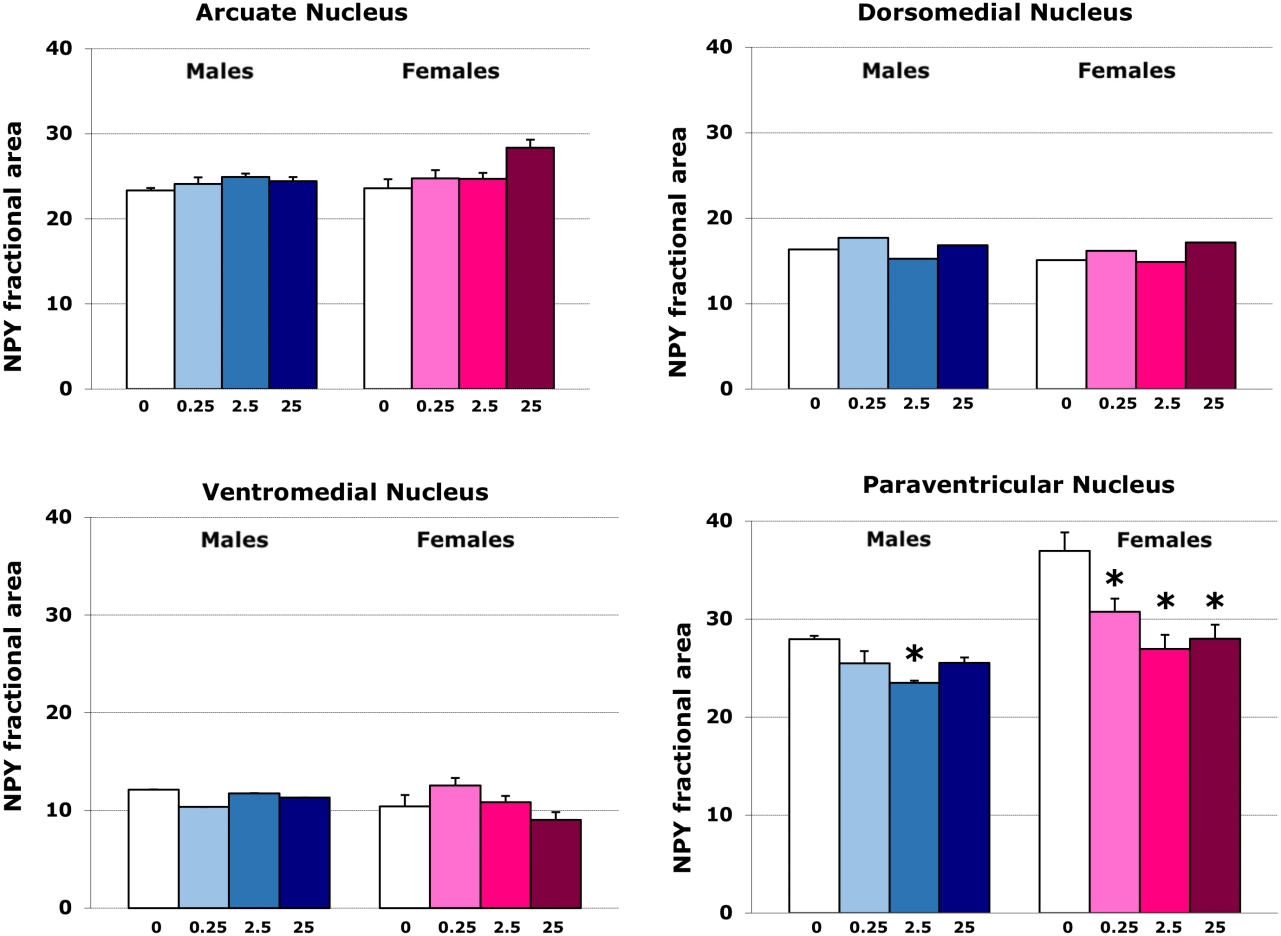
Quantitative analisis of variations of NPY immunoreactivity in ARC, DMH, VMH and PVN. Histograms represent the percentage of area covered by the immunoreactivity for NPY (fractional area) in different hypothalamic nuclei (arcuate, dorsomedial, ventromedial, and paraventricular nucleus). The only significant variations are in the PVN, with a general decrease of the immunoreactivity in TBT treated mice. This decrease is significant in males with the intermediate dose (2.5) and in females with all the doses. Females have a significant higher value of fractional area than males.

**FIGURE 4:**
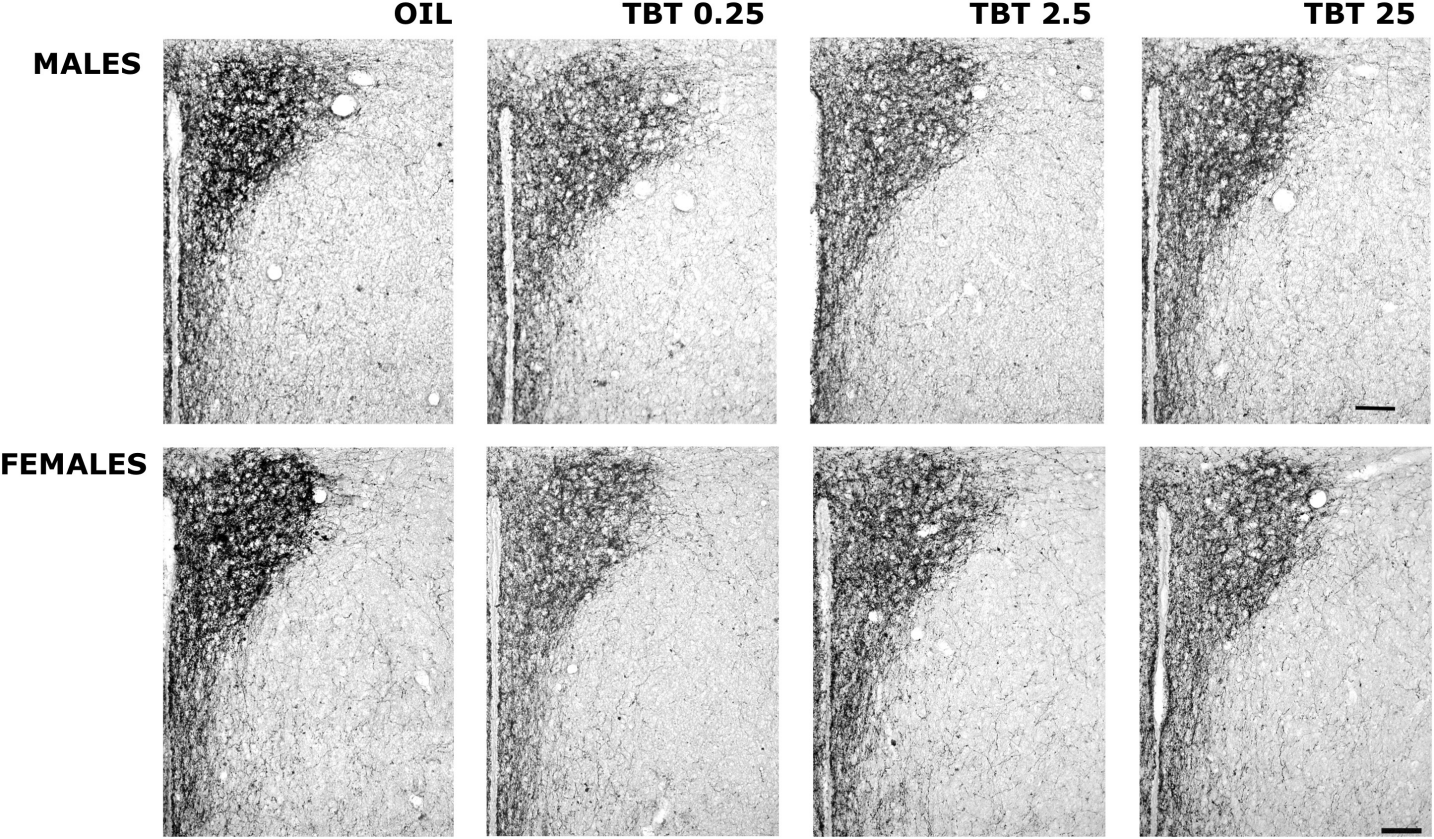
Variations of NPY immunoreactivity in the PVN. Photomicrographs illustrating the variations in the NPY immunoreactivity in the PVN of male and female mice treated with OIL or with TBT. In these representative photomicrographs it is possible to observe the reduction of the density of immunoreactive fibers in TBT treated mice.

Control (Figures 3 and 4) animals displayed a pattern of NPY immunoreactivity similar to that already reported (Bo et al., 2016; Marraudino et al., 2021). In particular, as usual in not colchicine‐treated animals, we observed only a few positive cell bodies, whereas a large number of positive fibres was present along the whole hypothalamus. These were particularly dense within the PVN and the ARC nuclei, but they were also abundant within the suprachiasmatic, supraoptic and DMH nuclei. Other regions, for example the VMH, had less dense immunopositivity. Qualitative inspection of the stained sections revealed differences between treated and control mice. We have, therefore, quantitatively analysed NPY immunoreactivity within nuclei that are involved in the food intake controlling circuit: PVN, DMH, ARC and VMH.

Our statistical analysis showed some differences between treated and control animals that were limited to specific hypothalamic nuclei.

The statistical analysis did not reveal significant differences between experimental groups in ARC (*F*_[7,30]_ = 1.457, *p* = 0.232; Figure 4a). In DMH, two‐way ANOVA analysis showed a statistically significant effect of treatment only (*F*_[3,30]_ = 3.853, *p* = 0.023), with no effect of sex (*F*_[1,30]_ = 1.654, *p* = 0.211) or of the interaction between sex and treatment (*F*_[3,30]_ = 0.584, *p* = 0.631). The multiple comparison analysis showed an increase in NPY‐ir in the DMH of females treated with the higher TBT dose, although this increase was marginal (*p* = 0.061).

In VMH, two‐way ANOVA analysis highlighted a statistically significant effect due to the interaction between sex and treatment (*F*_[3,30]_ = 3.197 and *p* = 0.042; Figure 4c), but no effect of sex (*F*_[1,30]_ = 1.270, *p* = 0.271) or treatment (*F*_[3,30]_ = 1.428, *p* = 0.260). The multiple comparison analysis did not reveal any significant difference.

Finally, in the PVN (Figure 4) two‐way ANOVA analysis showed a significant effect of sex (*F*_[1,28]_ = 38.687 and *p* < 0.0001) and of treatment (*F*_[3,28]_ = 14.454 and *p* < 0.0001), with no effect of the interaction between the two variables (*F*_(1,3)_ = 2679). Comparison between sexes revealed a sexual dimorphism in control animals: control females showed a higher amount of NPY fibres than control males (*p* = 0.001). In both sexes, treated animals showed a reduction in NPY immunoreactivity; however, in males, this reduction was significant (*p* ≤ 0.01) only for the intermediate TBT dose 2,5 μg/kg/day, whereas in females all TBT doses induced a significant decrease (*p* ≤ 0.05) of NPY fibres in comparison with the control group (Figure 3 and 4).

## 4 DISCUSSION

Obesogens are molecules that interfere with the homeostasis of the fat tissue, inducing differentiation of more fat cells and deposition of more fat droplets (Grun et al., 2006; Kirchner et al., 2010). It was supposed that these molecules (including organotins and therefore TBT) act only at the peripheral level. However, it is well known that several neuronal circuits are involved in fat tissue regulation and energy expenditure (e.g. circuits controlling food intake and circuits controlling metabolism; Gao & Horvath, 2007, Morton et al., 2006) and, therefore, could be targets for these molecules. The possibility of acting against multiple targets (i.e., peripheral and central) forms the basis of defining MDCs (Heindel et al., 2015, 2017; Heindel & Blumberg, 2019; Marraudino et al., 2019). We previously determined that TBT was directly acting at the central nervous system (Bo et al., 2011), using c‐fos immunohistochemistry after oral TBT administration: we observed a significant increase in c‐fos expression 90 min after TBT treatment, only at the level of ARC, the hypothalamic nucleus where NPY and POMC systems originate. Other studies demonstrated effects of TBT on neurons in vitro (Mitra et al., 2014; Nakatsu et al., 2009; Yamada et al., 2010), as well as alterations of mRNA transcripts encoding for hypothalamic hormones (*TRH*, Decherf et al., 2010; *NPY*, *AgRP*, *POMC* and *CART*, He et al., 2014) in vivo. The effects of organotins (including TBT) on the brain were recently reviewed (Ferraz da Silva et al., 2018). Recently, the peripheral nervous system (dorsal root ganglia) has been determined as a target for TBT (Fross et al., 2021). In our previous immunohistochemical studies, we demonstrated that chronic exposure to TBT in adulthood regulated the activity of both NPY and POMC systems (Bo et al., 2016; Farinetti et al., 2018; Marraudino et al., 2021).

We know that EDCs, as well as MDCs, may elicit two kinds of responses at the level of the central nervous system. They may have transient (activatory) effects altering neuroendocrine responses in adult animals (Graceli et al., 2020), or they may affect (in many cases in a sex‐biased manner) the formation of specific neuronal circuits during development (organisational effect, Street et al., 2018). Developmental effects are of concern, because they are often promoted by a much lower dose than the one considered safe (no observed adverse effect level; NOAEL) in adults and persist throughout lifetime.

In the present study, we focused on the organisational effects of perinatal exposure to different TBT doses below NOAEL. Both food intake and body weight were increased after TBT treatment. However, the effect on body weight was transient, thus we observed a general decrease in feeding efficiency in TBT‐treated animals. We observed a decrease of pups' body weight with the lower dose of TBT (0.25 μg/kg/body weight/day) limited in males to P12, while it persisted until adulthood in females. Interestingly, another study demonstrated that pups from TBT‐treated pregnant females had lower BWG than controls, albeit at a much higher dose (10 mg/kg/body weight/day, Cooke et al., 2008). This discrepancy may be due to the poor TBT transfer through the milk, which resulted in much lower dose in neonates than that in our mice. Neonatal TBT exposure may be particularly interesting, because satiety‐related signalling pathways in mice develop between P4 and P16, characterized by a surge in leptin levels (Ahima & Hileman, 2000). Since, in rodents, TBT transfer through the placenta is high, while its presence in the milk is minimal (Cooke et al., 2008), we chose to use prenatal indirect exposure to TBT through oral administration in mothers followed by postnatal direct treatment of pups until weaning. In these conditions, the effect of our treatment on body weight was transient, indicating that, similar to neonatal overfeeding (Sominsky et al., 2017), the effect of TBT treatment may be compensated in adults. Interestingly the most effective dose in males was the intermediate dose of 2.5 μg/kg body weight/day, whereas, in females the most effective dose was the lower one (0.25 μg /kg body weight/day), highlighting a different sensitivity to TBT between sexes.

Given the ability of TBT to activate some nuclear receptors, such as retinoid X receptors (RXRs) and PPARγ that are involved in adipogenesis (Nakanishi, 2008), it was expected that TBT may increase the incidence of obesity (Grun, 2014; Grun & Blumberg, 2006) and it is, thus, currently considered as a potent inducer of adipogenesis in vertebrates. In vitro studies demonstrated that exposure to TBT stimulated the expression of adipocyte differentiation markers (Inadera & Shimomura, 2005), and predisposed murine and human multipotent stem cells to undergo adipogenesis (Kirchner et al., 2010). In the present study, the lower dose of TBT treatment induced a significant increase in subcutaneous fat (which has positive metabolic effects, Tran et al., 2008), limited to males, while no changes were observed in other fat depots, or in females. These data suggest that perinatal exposure to this compound may induce susceptibility to metabolic disorders later in life, eventually when individuals are exposed to certain lifestyles (Heindel et al., 2015).

Peripheral alterations were accompanied by limited organisational effects on the hypothalamic NPY circuit. In fact, we observed a significant decrease in NPY immunoreactivity in the PVN of females exposed to all TBT doses, and of males exposed only to the intermediate dose. Cytotoxic action of TBT on neurons was demonstrated in vitro in mixed neuronal‐astrocytic primary cultures (Oyanagi et al., 2015), in primary hypothalamic cultures (Mitra et al., 2014), and in cultures of immature cortical neurons (Yamada et al., 2010), while in vivo studies showed alterations in different hypothalamic peptidergic circuits (Bo et al., 2016; Decherf et al., 2010; Farinetti et al., 2018; He et al., 2014; Marraudino et al., 2021). Differences in TBT doses, age and way of administration between studies prohibit direct comparisons between in vitro and in vivo studies.

Our results confirmed that the NPY system is a target for the central action of TBT administered during the pre‐ and postnatal period. However, here, the effects of exposure to TBT were limited to the fibres reaching the PVN (with a sex difference in the most effective dose), while other hypothalamic nuclei involved in the control of food intake and males did not show any significant change. This is in partial contrast to chronic exposure to TBT in adulthood (Bo et al., 2016; Marraudino et al., 2021) that induced a reduction in NPY immunoreactivity in the whole hypothalamic system. However, the two effects are profoundly different: in our previous experiments we have observed an activatory effect on a stable circuit, which will probably return back to the original state when the external perturbation (i.e., the TBT) is removed. In contrast, here we have essentially observed an organisational effect on the NPY innervation of the PVN, whose effects could be revealed later in life with unpredictable consequences on the regulation of the hypothalamus‐hypophysis axis. In fact, the NPY fibres from the ARC are contacting, in the PVN, the TRH (Diano et al., 1998; Eva et al., 2020; Fekete et al., 2002; Légrádi & Lechan, 1998) and CRF neurons (Li et al., 2000), thus controlling the two axes involved in the regulation of energy expenditure. A decrease in the NPY innervation of these two systems could have a major impact on the body's energy balance and on the susceptibility to different conditions during life.

Moreover, the slight sex difference we observed in the NPY system could be obviously due to an effect of sex hormones. To test this hypothesis, it will be necessary to repeat these experiments with gonadectomised mice.

## 5 CONCLUSION

In conclusion, our data suggest that TBT exposure during perinatal life may have long‐term effects that could be critical for the development of adult disease (obesity, metabolic syndrome). In fact, adult exposure to EDCs is certainly an important factor, however, focus on the fetus and/or neonate is of primary concern, since developing organisms are extremely sensitive to perturbations by chemicals with hormone‐like activity (Street et al., 2018). In fact, the protective mechanisms that are available in the adult are not fully functional in the fetus or newborns. In addition, the developing organism has an increased metabolic rate compared to adults that in some cases may contribute to increased toxicity. In particular, the alteration of NPY innervation of the PVN may interfere with the control of two important endocrine axes (ie. hypothalamus‐hypophysis‐thyroid and hypothalamus‐hypophysis‐adrenal axis) involved in the regulation of metabolism. Further studies (i.e. double staining with NPY and POMC) are necessary to elucidate whether other neural circuits may be affected by perinatal TBT exposure.

## AUTHOR CONTRIBUTIONS

GP and EB designed and performed the experiments, analyzed data and wrote the paper; B.B., A.F. and M.M. performed the experiments; S.G. and G.P. revised the manuscript, designed experiments, analyzed data, and wrote and supervised the paper.

## Data Availability

All the data are available from the authors upon reasonable request.
